# Life-Writing From Medicine: Biographies and Memoirs of Physicians

**DOI:** 10.15694/mep.2018.0000196.1

**Published:** 2018-09-05

**Authors:** Jacek Mostwin

**Affiliations:** 1 Johns Hopkins Medical Institutions

**Keywords:** life-writing, biography, memoir, physician writing, professional development, medical humanities

## Abstract

This article was migrated. The article was marked as recommended.

Life-writing is an increasingly recognized genre including, among other forms of expression, biography, memoir, journals, diaries and letters. In addition to providing a source of information about potentially significant historical and social dimensions of medicine, the life-writing of physicians also offers the public a greater sense of the writers’ inner lives and provides a resource of wisdom and companionship to other physicians and health care professionals across time and various stages of personal and professional development. This kind of life-writing, when considered alongside patient accounts, can further contribute to what Anne Hunsaker Hawkins has called “a medicine that is truly human”.

## Presentation

*I’d like to thank Meg Chisholm and Jonathan McFarland for their kind invitation to participate in this meeting. It’s an honor to be here among such distinguished speakers. So thank you very much. By way of introduction, I should say that I have a long background in clinical urology, with involvement in surgery, research, education and ethics, and somewhere along the way I developed an interest in patient memoir and narrative, and then eventually, in physician memoir and narrative. This interest has grown and will be the focus for my presentation today..*

In her landmark 1993 book, *Reconstructing Illness: Studies in Pathography,* Anne Hawkins introduced a novel way of describing published personal accounts of patients about their illness experience, a *genre* she entitled *pathography*:

*.. a form of autobiography or biography that describes personal experience of illness, treatment, and sometimes death. By writing pathographies, patients not only restore the experiential dimensions to illness and treatment but also place the patient at the very center of that experience. ( Hawkins, 1993)*

As clinicians, we have become more familiar with these voices, even if they do not always have a prominent role in the formality of clinical encounters. We do not need to overemphasize these patient accounts today, as you are likely somewhat familiar with the *genre*, especially given Rita Charon’s advocacy for *Narrative Medicine ( Charon, 2008).* What you may be less familiar with, however, are the remarks Hawkins added to the conclusion of her book in 1993 regarding writing from the other side of the clinical relationship: the biographies and memoirs of practitioners:

*Another voice we need to hear is that of the physician. This may seem a paradoxical statement at the end of a book that so insists on returning the patient to the medical enterprise and so often contrasts the patient’s voice to that of medicine. But the “physician’s voice” I am referring to [ .. is] the voice of the individual who is inevitably lost in that impersonal professional voice. We need to hear from them. [ .. ]*

*We need more writing that conveys the inner reality of what it is to be a physician in today’s technological medical system. Only when we hear both the doctor’s and the patient’s voice will we have a medicine that is truly human.* ( Hawkins, 1993)

**Figure 1.**
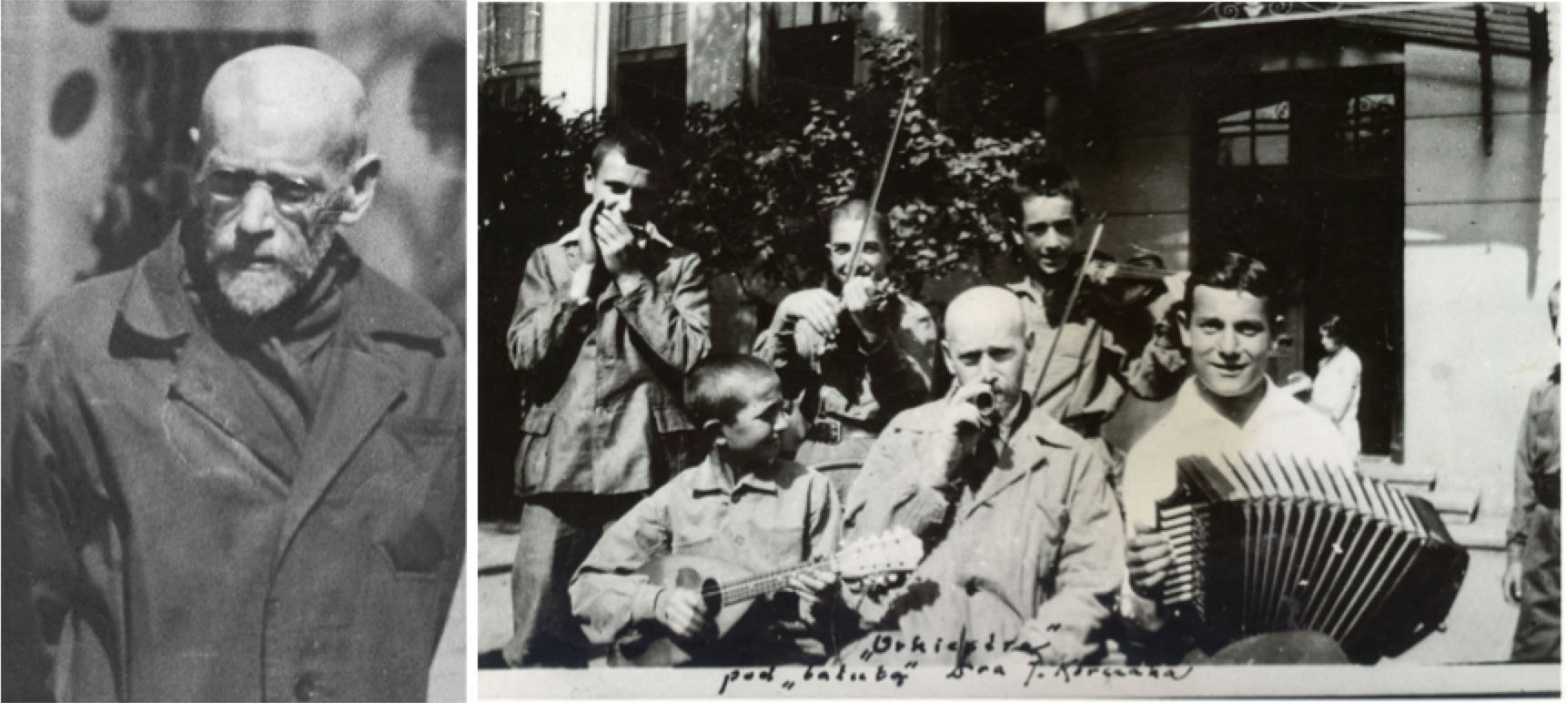
(L) Janusz Korczak in front of the Orphan’s House in 92 Krochmalna St., Warsaw, ca. 1938-39, photo courtesy of the Korczakianum Centre for Documentation and Research in Warsaw. Ref: Gliński, Mikołaj. 12 Things Worth Knowing About Janusz Korczak. Culture.pl #Language and Literature. Published Sep 16, 2016. url: https://culture.pl/en/article/12-things-worth-knowing-about-janusz-korczak (R) Janusz Korczak and the children in front of Orphans Building in Warsaw at 92 Krochmalna street (before 1939) Ref: 2101: The Year of Janusz Korczak, Tourwarsaw.wordpress.com Mar 11, 2012. url: https://tourwarsaw.wordpress.com/2012/03/11/2012-the-year-of-janusz-korczak-in-poland-11-2/

It is these personal voices that I wish to consider today. One such doctor’s voice was that of Janusz Korczak ( Figure 1). In 1942, confined to the Warsaw Ghetto in occupied Poland he wrote: *“Everyone should know how to sketch in pencil what he wants to retain in memory. Not to be able to do that is to be illiterate.” ( Korczak, 2003)*

And that is what we are addressing at this conference: this broad sense of literacy in medicine. “ *No wonder* [Korczak went on in *Ghetto Diary*] *, that the memoirs are incomprehensible to the reader. Is it possible to understand someone else’s reminiscences, someone else’s life?*” Yet, through the writing of these physicians, whether living or dead, we do seek contact with their lives, whether from mere curiosity, or to find wisdom or perhaps seek companionship from a colleague who might understand us.

Janusz Korczak was born Henryk Goldszmyt into an assimilated Jewish family in 1878 in Warsaw. He was torn between literature and medicine, but chose a life of service that medicine offered; he became a noted pediatrician, he continued to write and he became a popular writer of children’s books. In mid-life he gave up his successful practice to run a Jewish orphanage without salary, still writing and conducting radio programs. During WW II, the occupying Germans forced the orphanage into the cordoned portion of Warsaw known as the ghetto; he moved there with his children. In April 1942, the ghetto was being liquidated, its inhabitants sent to death camps for extermination. He stayed with his children rather than accept offers of freedom provided because of his celebrity. He kept a diary, which was hidden, later found and published as *A Ghetto Diary,* the source of these earlier fragments. This is the kind of *Life-Writing* by physicians to which I am referring.

Virginia Woolf is credited with creating the term *Life-Writing*, now an accepted form of literary scholarship better known in the UK, where it has found a home in several university centers. I was fortunate to be a Visiting Scholar at one: the Oxford Centre for Life-Writing for periods during 2014-2016 to become more familiar with the genre and its study. ( https://www.wolfson.ox.ac.uk/what-life-writing)

**Figure 2.**
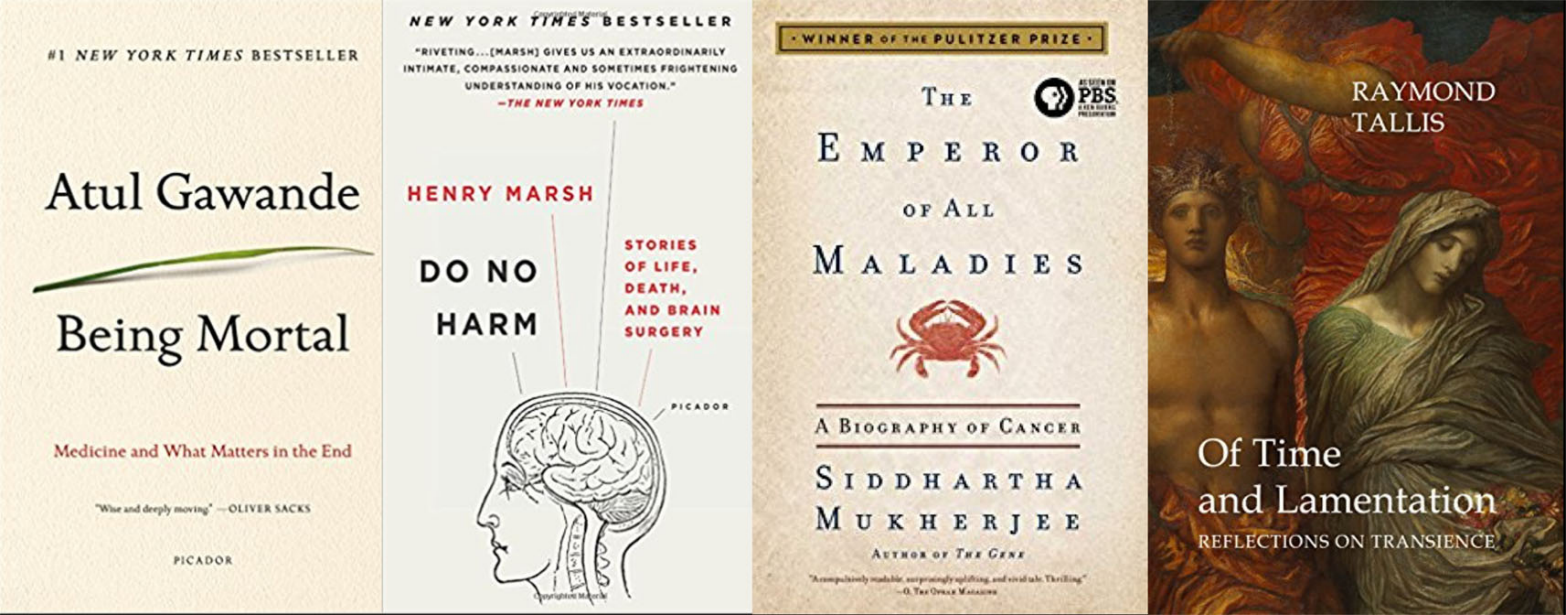
From author’s collection

Many physicians write, but not all physician writing is life-writing. Atul Gawande’s *Being Mortal ( Gawande, 2014)* has been praised for candor, personal disclosure, elegant style and a scholarly approach to end of life care. Siddhartha Mukherjee’s *Emperor of All Maladies* won the Pulitzer Prize in 2011 ( Mukherjee, 2017). In the U.K., Henry Marsh’s *Do No Harm* brought the inner world of neurosurgery and its limits to the public. ( Marsh, 2016). Raymond Tallis has been recognized for philosophical books about the roots and methods of medicine ( Tallis, 2017). These authors speak *ex cathedra,* remaining in their white coats. ( Figure 2)

These are big books that receive both awards and recognition that is well deserved. But I wish also to consider other voices, recalling Hawkins, *“the voice of the individual who is inevitably lost in that impersonal professional voice.”* A voice that brings u *s “more writing that conveys the inner reality of what it is to be a physician in today’s technological medical system,”* and sometimes, as it will turn out, before today’s technological system.

**Figure 3.**
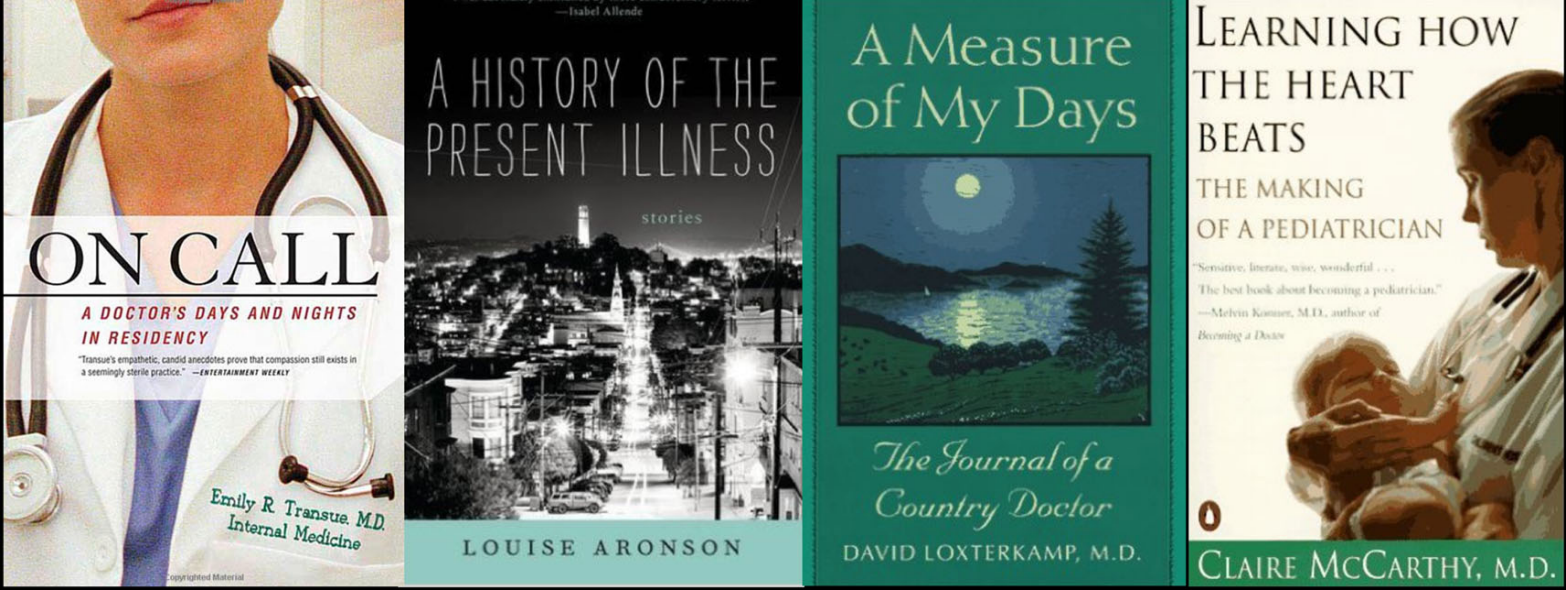
From author’s collection

Consider Louise Aronson’s *History of the Present Illness* ( Aronson, 2014) and Emily Transue’s *On Call ( Transue, 2013)* ( Figure 3) that tell familiar tales, recalling clinical encounters but carefully crafted, formally presented, absorbed more deeply by a reader in quiet moments than if heard passing in the hallway or over coffee. The written word endures with a gravity that the spoken word may miss. *Verba volent, scripta manent* [words are fleeting, writing endures].David Loxterkamp is a general practitioner in a small town on the Maine coast detailing the day-to-day in *A Measure of My Days* ( Loxterkamp, 1997). Claire McCarthy is an inner city pediatrician in Boston who wrote from her medical school and residency experiences at Harvard: *Learning How the Heart Beats: The Making of a Pediatrician* ( McCarthy, 1997), and later *Everyone’s Children: A Pediatrician’s Story of An Inner City Practice* ( McCarthy, 1998) Her writing also presents familiar clinical scenes but with moral lessons of the sort we know our students think about, but rarely have a chance to speak or write about. McCarthy’s writing, as much as the other physician writers here described, speaks to the reader’s heart from the front lines of medical work. ( Figure 3)

From preliminary searches, I estimate that some thousand or so such accounts have been published from 2010 through 2016. It is tempting to acquire these books, categorize them, look for patterns, and generate theories and structures. In medicine, we do that very well. We approach the unknown this way. But already in 1933, Alfred Korzybski, creator of the Theory of General Semantics, had warned us that: “.. a map is not the territory” ( Korzybski, 1995). So I think we should not be too hasty in our research, but rather dig deeply into one person’s life, one at a time.

**Figure 4.**
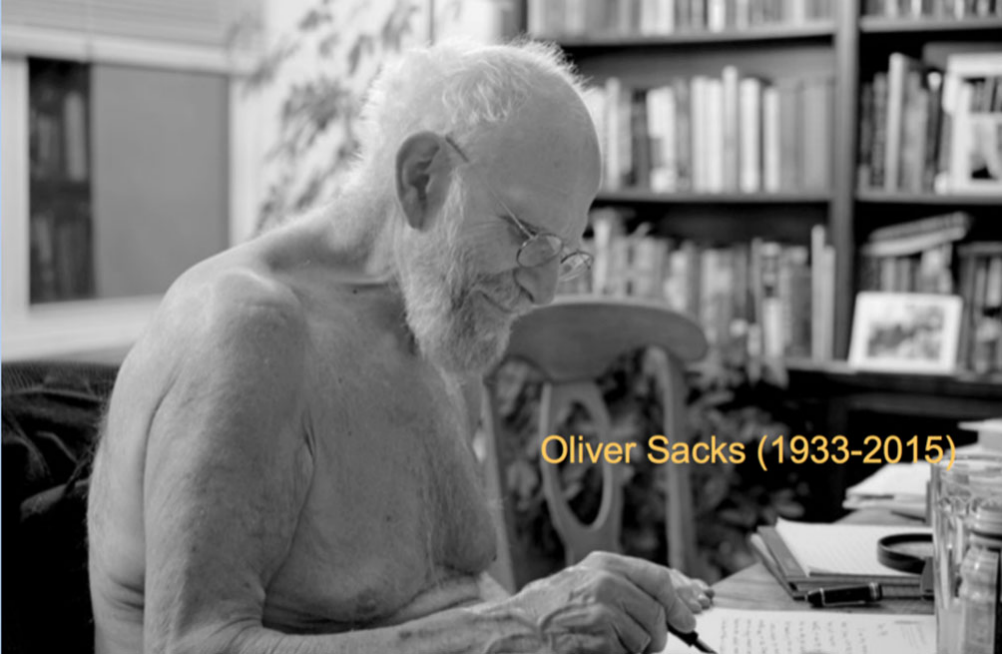
Oliver Sacks at his New York City home in 2015. Credit Bill Hayes Hayes, Bill. *Out Late With Oliver Sacks.* New York Times August 26, 2016. url: https://www.nytimes.com/2016/08/28/opinion/sunday/out-late-with-oliver-sacks.html?smid=pl-share

For Oliver Sacks ( Figure 4), the map was not the territory. He often went beyond succinct clinical summaries of the sort students and young doctors feel they must learn to create out of the jumble of subjective and objective information they obtain from personal encounters and reviews of objective data about their patients as they learn to master the writing of concise, distilled summaries. By contrast, those who sent Sacks patients for consultation sometimes dreaded the lengthy notes they received back, extensively detailing aspects of the patient’s life that many other doctors might find irrelevant, or as we would say in the U.S.: “non-contributory”.

**Figure 5.**
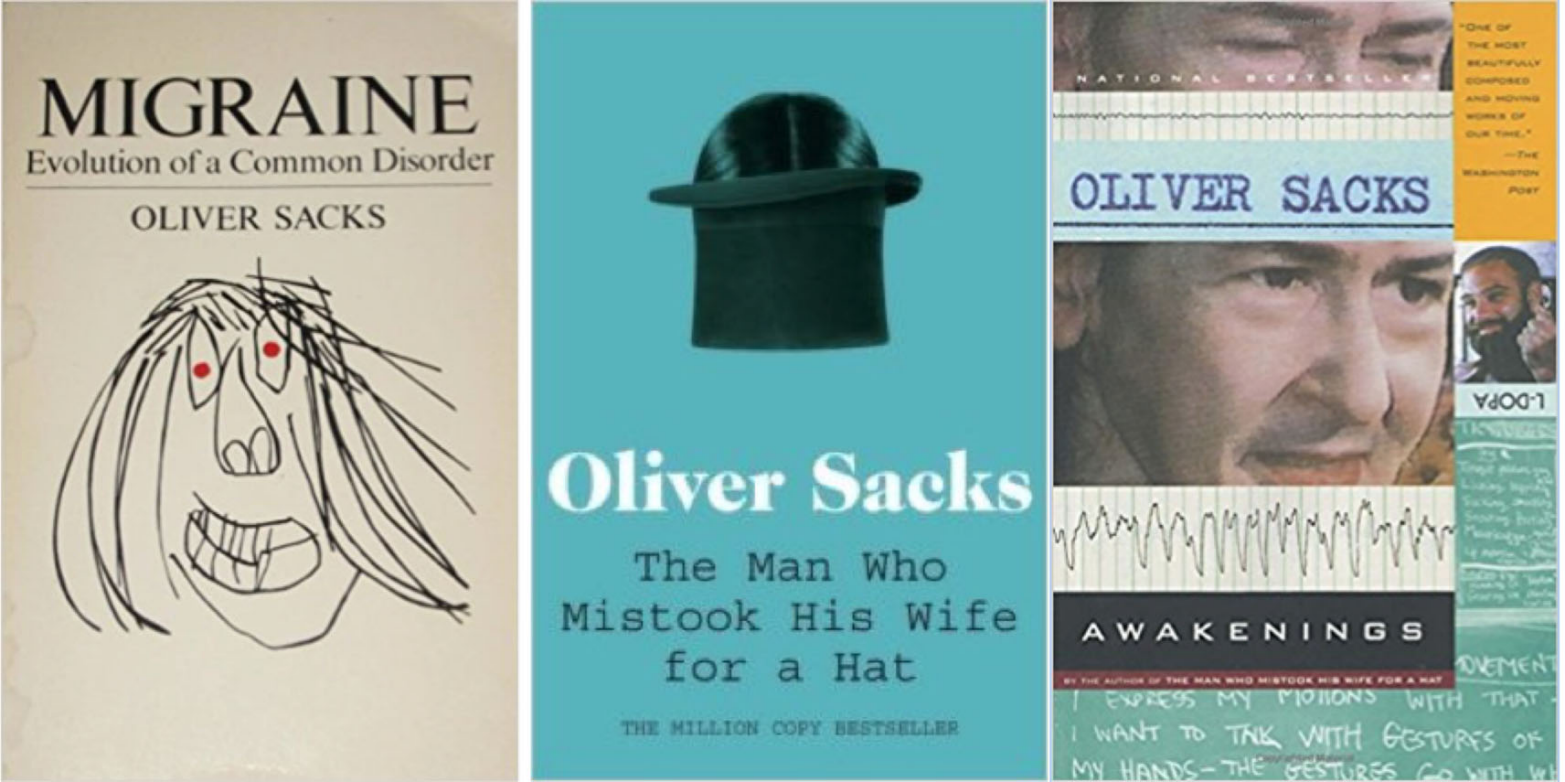
From author’s collection

Perhaps it was these non-contributory elements that led Sacks away from the map. He dove deeply into details of migraine and its equivalents, producing extensive clinical case reports, using as a model those cases of Edward Liveing written in 1867. Liveing’s book, as discussed in Sacks’ *Migraine* ( Sacks, 1970), is rich in cases and details, but Sacks found that the book and its methods had been all but abandoned by contemporary medicine. He then wrote up detailed clinical cases of comatose patients suffering from *encephalitis lethargica* that responded to dopamine in *Awakenings* ( Sacks, 1973), naming the chapters after the first names of the patients instead of cataloguing them with the names of clinical syndromes, as he had done in *Migraine.* David Wallace-Wells, writing in *New York* magazine, noted that the British poet W. H. Auden had read these earlier works, neither of which was especially successful when first published, and became his friend. “You’re going to have to go beyond the clinical,” he wrote to him. “Be metaphorical, be mythical, be whatever you need.” ( Wallace-Wells, 2012) Sacks then wrote *The Man Who Mistook His Wife For a Hat* ( Sacks, 1985), a light but personal and imaginative account of unusual neurological cases presenting in unusual ways. The *Man Who Mistook His Wife* made Sacks a celebrity. ( Figure 5)

Sacks did not write without criticism. Tom Shakespeare, a prominent British writer and disability advocate, criticized him for being “the physician who mistook his patients for a literary career.” ( Shakespeare, 1996) It would seem that his approach was not universally shared by all those whom he thought he was serving.

**Figure 6.**
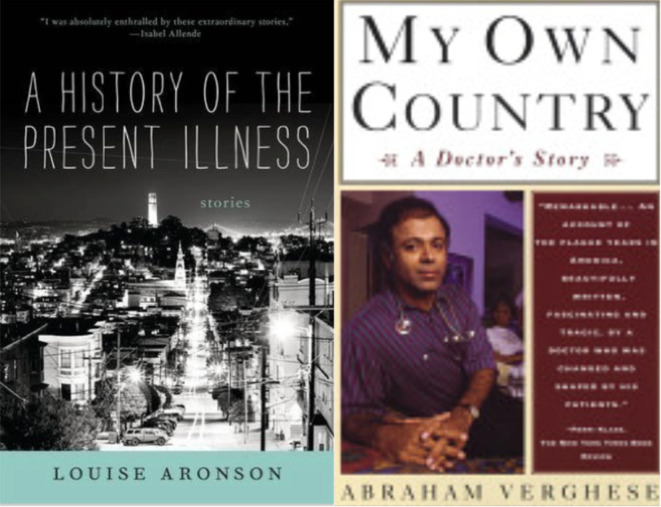
From author’s collection

In *The History of the Present Illness* ( Figure 6) *,* Louise Aronson sought to avoid this potential conflict by creating fictional composites of real patients. Others remove names and identifiers. We know they are telling us stories of real life that, despite whatever alterations, seem plausible. They must, else they would run the risk of straying too far from what drove Robert Lowell, in his poem *Epilogue*, to ask: “Yet why not say what happened? Pray for the grace of accuracy..” ( Lowell, 1978)

And so we read these works of those who say what happened, such as the personal vignettes of McCarthy and Transue. We read Abraham Verghese’s *My Own Country* ( Verghese, 1994) ( Figure 6) *,* lyricallycombining details of his patients’ lives with his own maturation in American medicine as a small town doctor from a talented Indian medical diaspora caring for the abandoned sons of great Southern families, returning home from America’s gleaming cities to die of AIDS.

**Figure 7.**
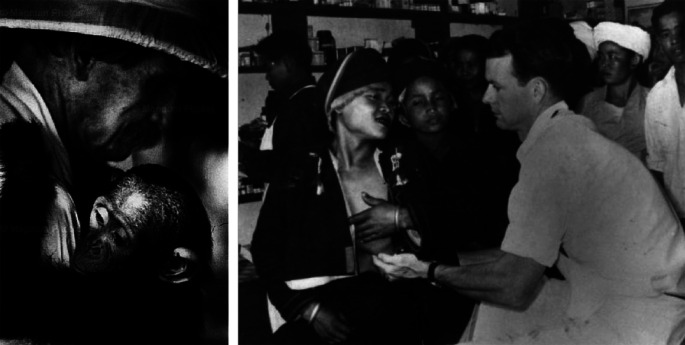
(L) photo from: Smith, W, Eugene, “Man of Mercy” LIFE, New York, Nov. 15, 1954. (R) Ref: O’Neill., Tim. *A Look Back - Tom Dooley Jungle Doctor, Tom Dooley, Succumbs to Cancer in 1961.* St. Louis Post-Dispatch, Jan 18 2014. url: https://www.stltoday.com/news/local/govt-and-politics/a-look-back-the-jungle-doctor-tom-dooley-succumbs-to/article_bec381d7-2c9c-5875-a6e1-8d7776342afd.html

Albert Schweitzer (1875-1965) and Tom Dooley (1927-1961) ( Figure 7) also found their own countries earlier in the 20 ^th^ century. Schweitzer in Equatorial Africa, Dooley in Southeast Asia. They both wrote extensively, Dooley’s life was cut short at the age of 34 by melanoma; Schweitzer lived to 90. They once sat together in Lambaréne to discuss what mattered in life. Schweitzer told him: “The significance of a man, Tom, is not in what he attains, but rather in what he longs to attain.” ( Dooley, 1960). Many conferences could be devoted to the lives and writings of these two men.

Albert Schweitzer, creator of the ethical theory of “Reverence for Life”, author of 24 books and edited collections of letters including the monumental *Out Of My Life and Thought* ( Schweitzer, 2009) author of 11 edited collections of this writings, and subject of 34 books about him was awarded the 1952 Nobel Prize for Peace, not, somewhat ironically, for Medicine. When in 2015 I asked a group of 90 first year medical students in my ethics class how many had ever heard of him before entering medical school, the number who had was 10%. How much were they missing by not knowing about him, let alone, not reading anything by him or about him? Or, I wondered, was I out of touch with the present, pre-occupied by nostalgia for the past? Why did Schweitzer matter to me? Maybe I was missing something?

Were these doctors humanists because of what they wrote or because of how they lived? Is it the literature they created or the lives they lived that matter more to us? We sense that their accounts are deeply human in perspective, giving us first hand descriptions filtered through a clinical intelligence that we as doctors understand and can often recognize as being similar to our own. Where exactly do they fit into literature, into medicine?

World War II left us many memoirs by military physicians at the front, but I will speak of three left by civilians.

**Figure 8.**
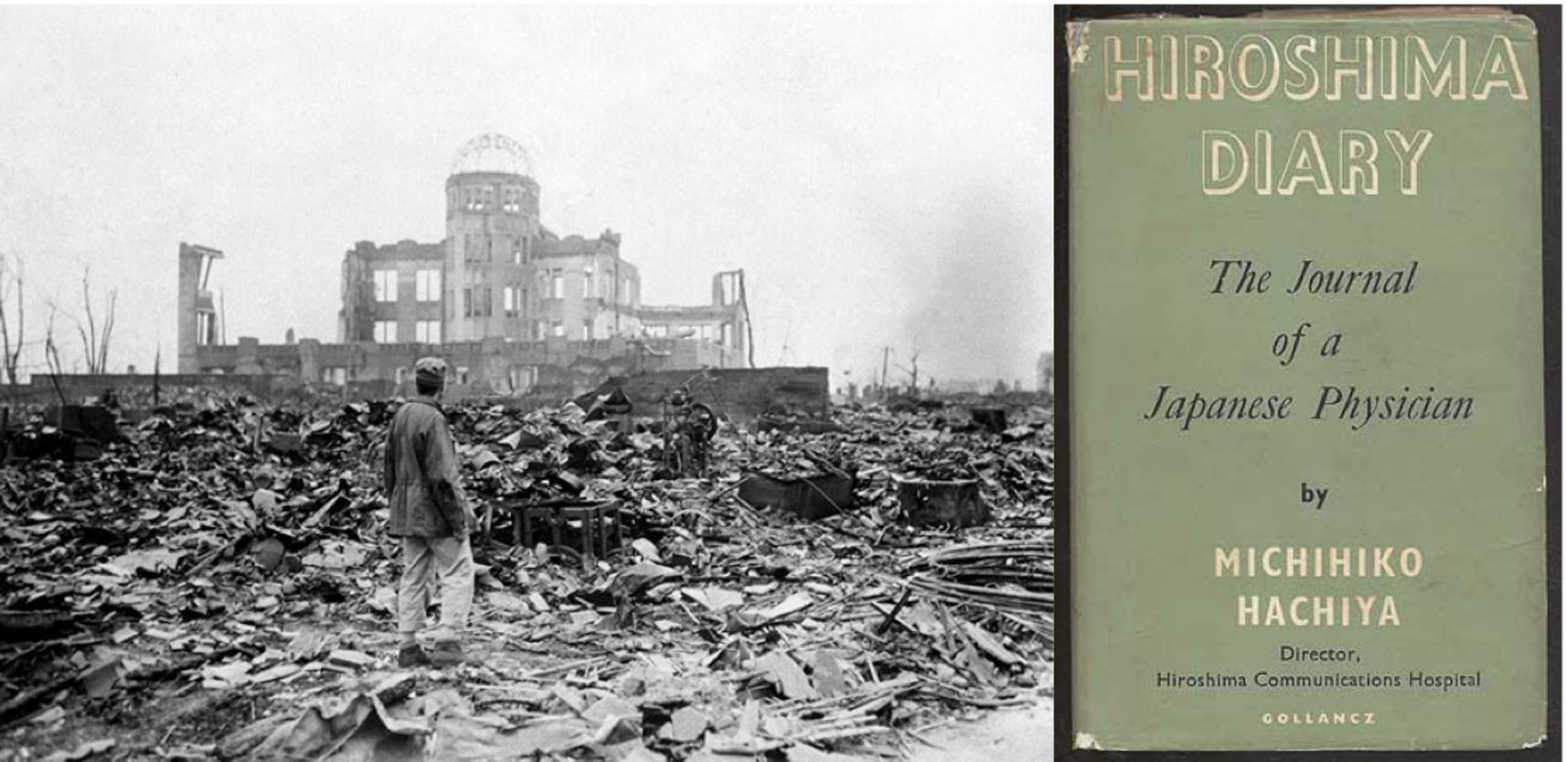
(L) photo: Diaz Alvarez, Enrique. *Los seis de Hiroshima.* Confabulario. Aug 29, 2018. url: http://confabulario.eluniversal.com.mx/los-seis-de-hiroshima/; (R) book jacket: author’s collection

*Hiroshima Diary: the Journal of a Japanese Physician* was written by Michihiko Ishida, Director of the Hiroshima Communications Hospital, from memory ( Ishida, 1955) ( Figure 8). He was present within the killing radius of the first atomic bomb. He converted partially destroyed buildings into a hospital without a ceiling, attending to burn victims and those dying of radiation sickness without knowing what he was treating, as the syndrome had never been clinically seen before. He cared for his wife and his colleagues and endured radiation sickness himself, all the while describing in a quiet way the destruction around him, the scenes he witnessed and the gestures of decency and care that surrounded his makeshift hospital until the Americans finally arrived. His diary was discovered by Dr. Warner Wells, an American Surgeon of the U.S. Atomic Bomb Casualty Commission, which had been created to study the effects of radiation on the body, but not to treat its victims. Wells taught himself enough Japanese to oversee a translation. I found it by accident on the WW II history shelf of Baltimore’s Enoch Pratt Free library. J. Robert Oppenheimer, director of the Manhattan Project that designed the atom bomb, who was himself reported to be uninterested in radiation’s biological effects on people or the value of physicians as scientists, wrote of it: “A simple and unpretentious account of compassion, sorrow and great courage..I read it through in a sitting, putting it down very rarely.”

In the European theater, while searching for books by Paul Tillich, I came across a memoir by Hans Graf von Lehndorff (1910-1987) : *Token of a Covenant: Diary of an East Prussian Surgeon 1945-1947* (von Lehndorff, 1963) *,* for which Tillich had written an introducti *on.* ( Fig 9a & 9b)

**Figure 9a.**
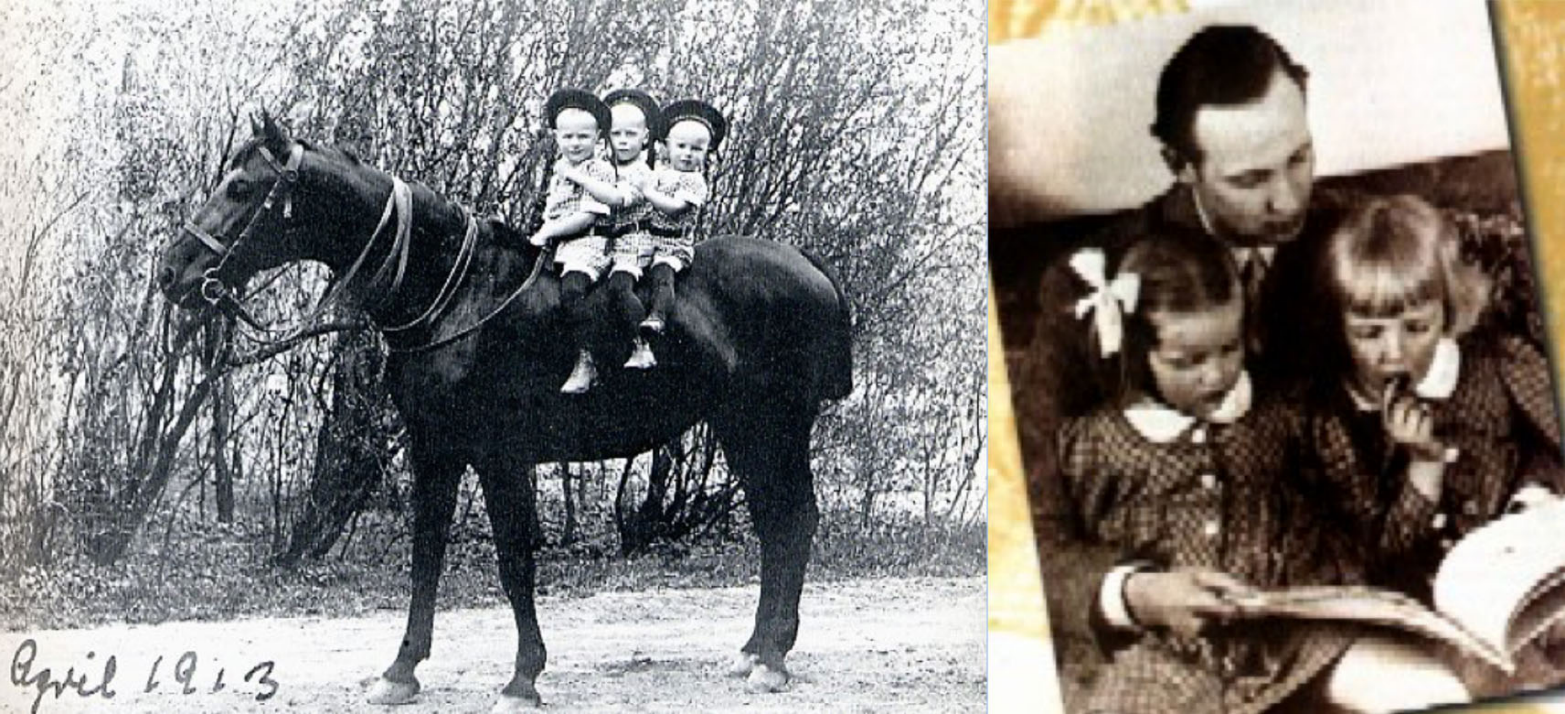
photo refs (L) url: http://www.aefl.de/ordld/Januschau/januschau1/januschau_1.htm (R) url: https://www.soundwords.de/mein-geliebtestes-a149.html

**Figure 9b.**
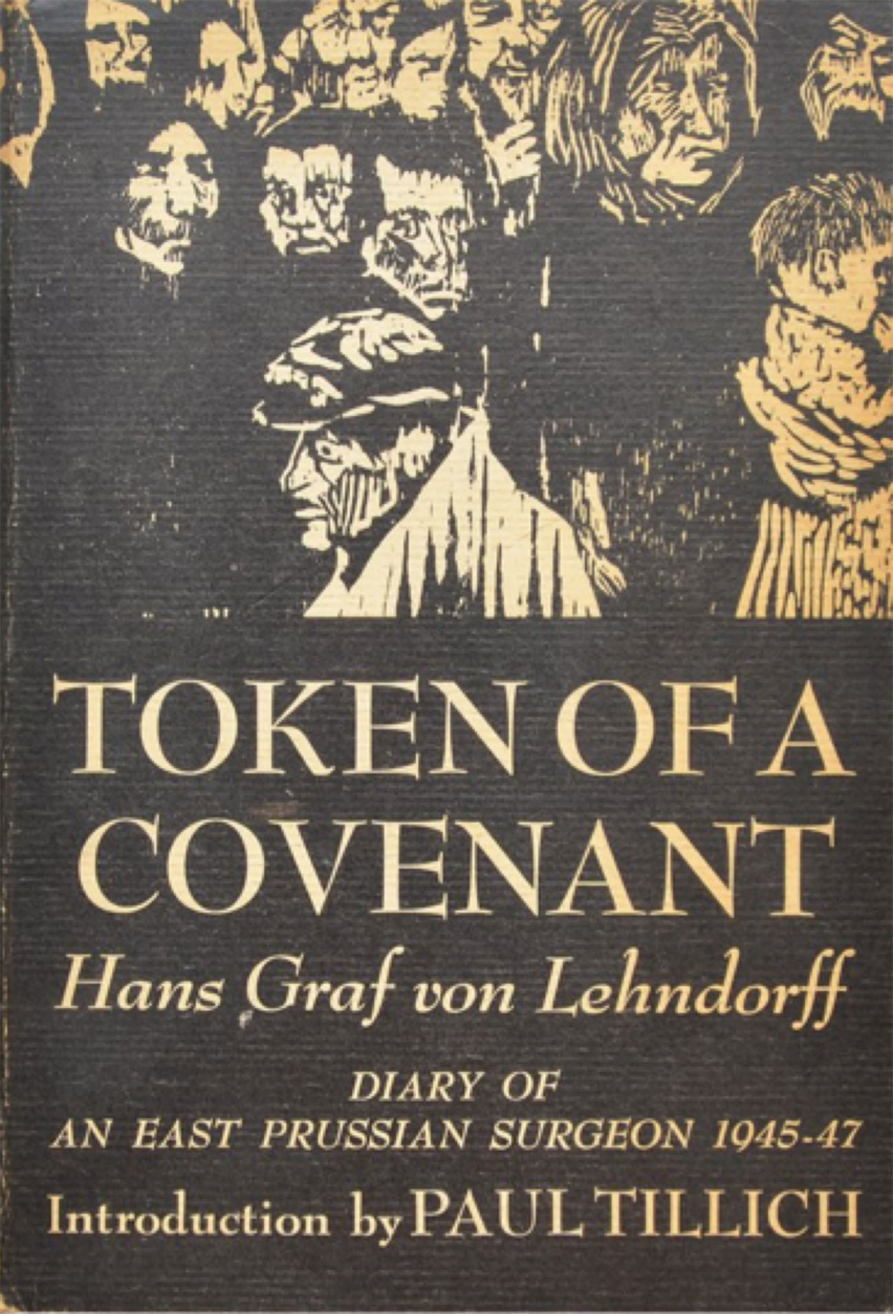
From author’s collection

Lehndorff, an aristocrat trained as a surgeon, was stranded in the remaining German sections of East Prussian, now northeast Poland, when the Russians began their final advance to Berlin at the end of World War II, exacting considerable retribution against the ethnic Germans who had remained there. Lehndorff was several times imprisoned, nearly shot, yet practiced medicine, performed operations with makeshift instruments, washed and re-used bandages, treated his captors, attended to friends and colleagues dying of typhus and malnutrition, occasional random shootings or beatings. He narrowly avoided death alongside his mother and brother who were shot by the Russians. In spite of everything, he managed to hold structured prayer meetings, he practiced medicine as best he could, he survived and finally escaped across the Western border of Poland on foot.

Wanda Półtawska, someone I don’t think may of you would know, was a teenager when she was arrested by the Germans in Poland ( Półtawska, 2013) ( Figure 10a & 10b). She was eventually sent to the concentration camp at Ravensbruck that would gain infamy for subjecting women to human experimentation as was later revealed during the hearing in Nuremberg.

**Figure 10a.**
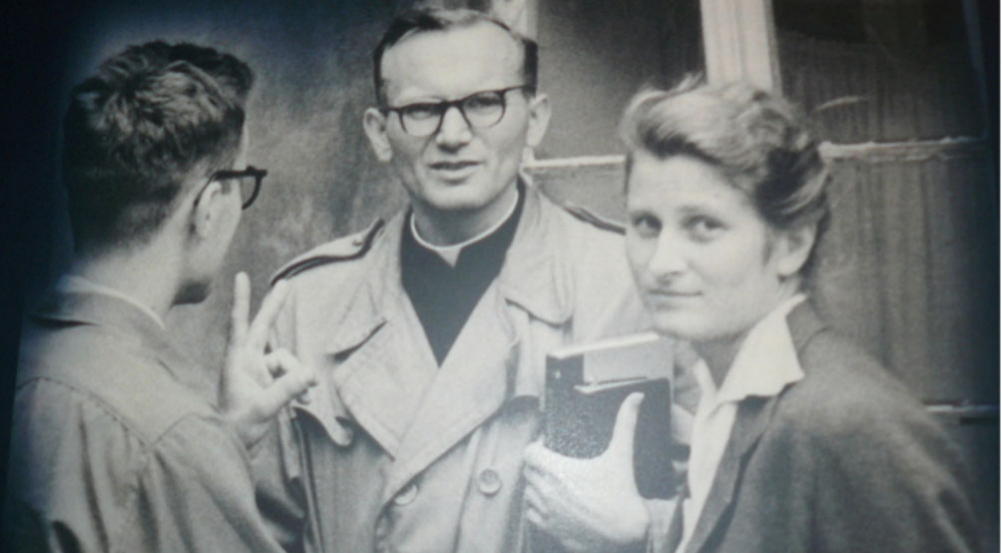
Wanda Poltawska with Karol Wojtyla The Historical House. August 19, 2016 url: http://thehistoricalhouse.blogspot.com/2016/08/wanda-potawska.html

**Figure 10b.**
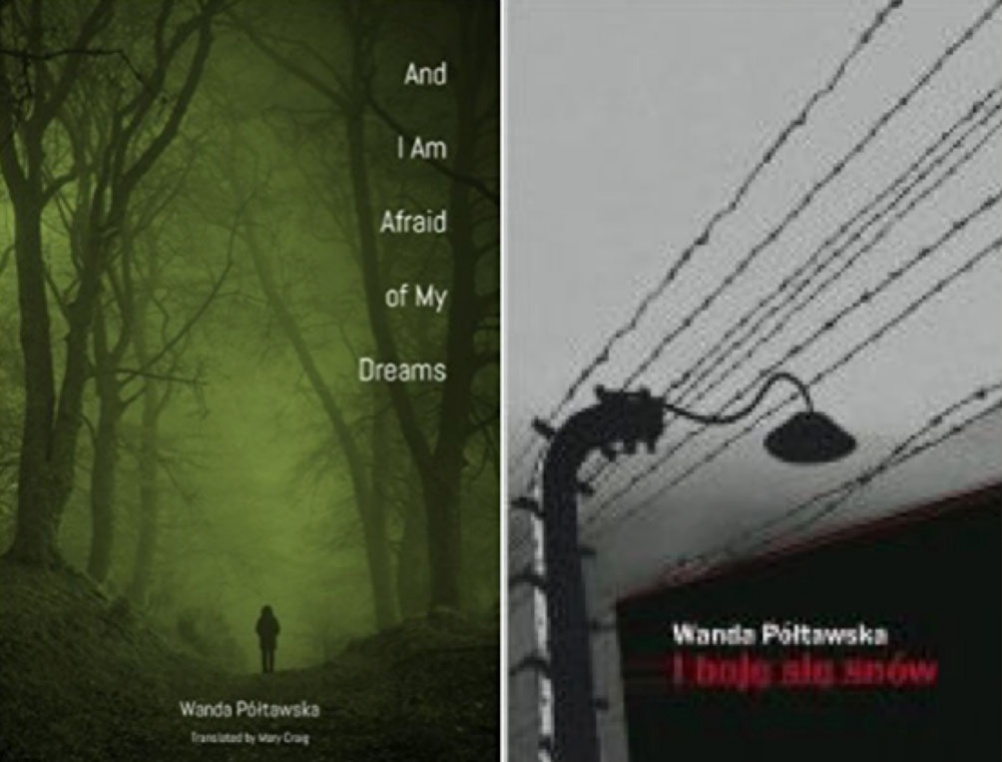
From author’s collection

Many of these “experiments” involved the breaking of the bones of lower extremities, removing segments of bone, or creating open, infected wounds to study healing, a form of “scientific” medicine whose history only became known after the war. Półtawska survived Ravensbruck, the camp was liberated; she walked home, studied medicine and became a psychiatrist. She began providing medical and psychiatric care to concentration camp survivors. She wrote her memoirs after being persistently haunted by the memories of what she had experienced.

These are memoirs no one should have to write. But that is our world.

The final person I wish to consider sometimes seems to have lived in another world, conjured as if from a forgotten time, far removed from the horrors described in the war memoirs just considered. His life-writing invites us to a wider realm of medical literacy beyond what we usually think of as medical literature. It is writing at home in the world of letters, sharing ways of living, conveyed by a thoughtful person who happens to be both a participant and a privileged observer of the human condition: a doctor. With a little imagination, we might consider him to be one of us.

**Figure 11.**
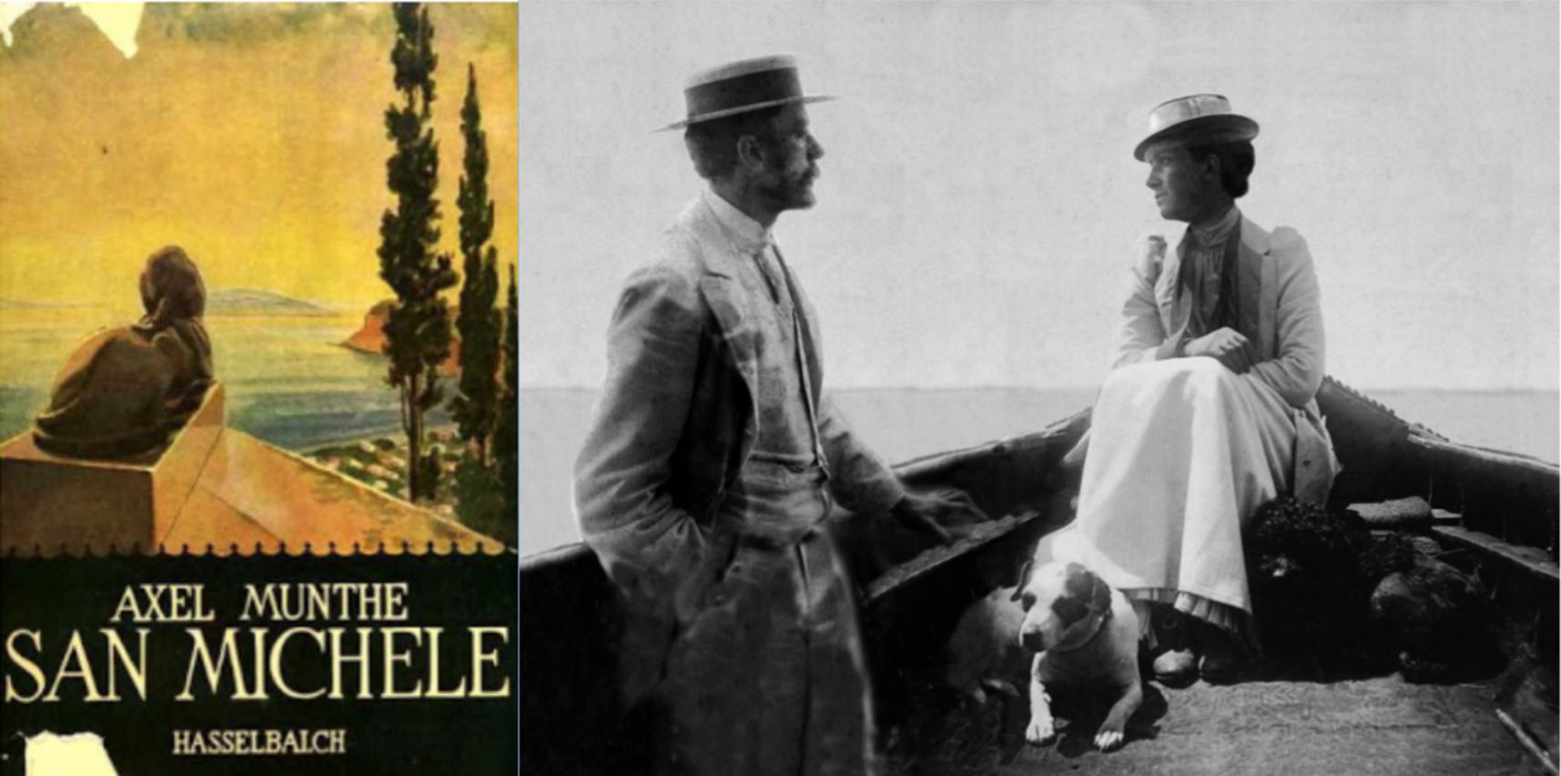
(L) Authors collection. (R) Axel Munthe and Crown Princess Victoria on Capri, Italy, the late 1890s. Ref: *Victoria of Baden - A forbidden Love.* November 3, 2017. url: https://www.historyofroyalwomen.com/the-royal-women/victoria-baden-forbidden-love/

Axel Münthe (1857-1949) was a Swedish physician whose practice and readership stretched across Europe and Great Britain. I first met him in the pages of his final book, The *Story of San Michele* ( Münthe, 1929) ( Figure 11), named after the sanctuary he resurrected from the ruins of a Roman imperial villa on the island of Capri in the Bay of Naples. It is a memoir constructed of vignettes from his life and clinical work, conveying his sympathies for people and animals, humoring his sense of the whimsical, the ironic and the fantastical, and his deep, humane compassion for life. It is a voice that, as Auden encouraged Sacks to be, also tends toward the metaphorical and mystical, but a voice of sympathy and wisdom, romantic, yet firmly grounded in reality.

One could imagine entering his home and engaging in dialogue about life and medicine. I read and re-read his earlier works, *Letters from a Mourning City* ( Münthe, 1899) about cholera in Naples, *Memories and Vagaries* ( Münthe, 1937) about his early experiences with the sick, *Red Cross and Iron Cross* ( Münthe, 1930) about his experiences as a WW I battlefield surgeon. Once he had mastered his craft, he became what he most wanted to be: his own man and a trusted companion to others. Among his patients was Victoria, Crown Princess, and later Queen, of Sweden. Perhaps on his boat or from the veranda of his villa, overlooking the azure blue waters of the Mediterranean, Münthe listened, nodded, reassured and provided consultations and sometimes interventions. At other times, he snuck away from his life of charm and glamour to attended the poor of Capri who revered him, or the Neapolitans dying of cholera in the poor districts, without regard for his own safety, and the similarly poor Italian servants in Paris, mindful of the indignities, humiliation, servitude and disenfranchisement, which their poverty and lack of education had forced upon them.

Though raised as a Northern Protestant, he had sympathy for the populist Catholicism of the Mediterranean that gave hope to his patients and inspired such religious orders as the Little Sisters of the Poor, whom he protected anonymously. Yet he was self-effacing and his life was not without its own personal sorrow. His marriages were unhappy. His various families were fragmented. He lost parts of his feet climbing the Alps, suffered recurrent hemorrhages from pulmonary tuberculosis, and lost vision completely in one eye and nearly completely in the other, making it impossible for him to tolerate the bright sun of Capri. Yet he was deeply loved by many who were the beneficiaries of his medical presence or saw in him a model for humane medicine.

Along with Schweitzer, Dooley, and the few selected others mentioned in this brief presentation, an entire conference could be devoted to their lives and work. Münthe is only one of many whose life-writing has left us a legacy and a sense that we are welcomed, even entitled, to consider a variety of ways of being doctors, regardless of our specialties, even in an industrialized world. Perhaps it requires a special way of reading that only physicians might understand, having shared experiences similar enough to his own to allow us to see these possibilities, but we should not forget that Münthe’s literary success went far beyond medicine, that he was one of the most popular and translated writers of his time, widely read and appreciated.

**Figure 12.**
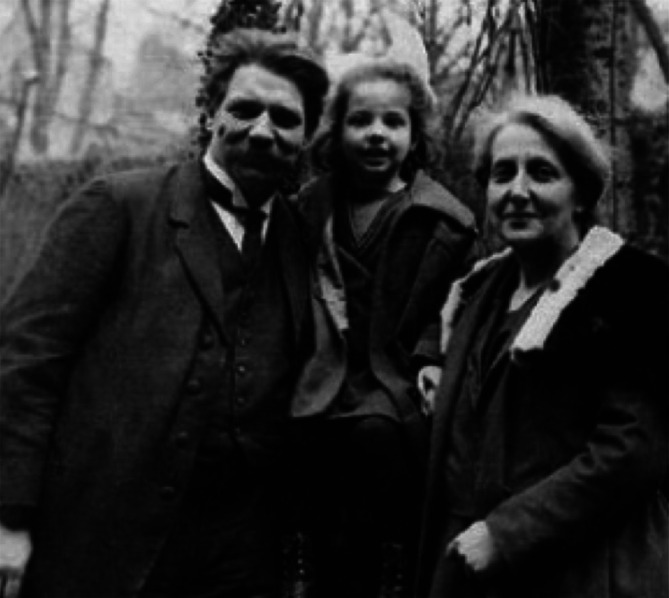
Albert und Helene Schweitzer mit Tochter Rhena. Ref: *Familie Schweitzer in Königsfeld.* url: http://www.albertschweitzer-haus.de/de/Albert-und-Helene-Schweitzer/Familie-Schweitzer-in-Königsfeld

My library of patient and practitioner life-writing has been growing. I would like to read many of the books I have found to satisfy my ambitions and perhaps to create structure out of disorder, but I am beginning to wonder whether I really need to do that. I have discovered the satisfaction that one obtains from immersion into the story of one life at a time in preference to what might appear to be a grander aggregate analysis. I am taking a clue from Albert Schweitzer ( Figure 12) when he wrote: “I have given up the ambition to be a great scholar..I want to be more simply a human..” ( Cicovacki, 2009)

## Take Home Messages

In addition to providing a source of information about potentially significant historical and social dimensions of medicine, the life-writings of physicians also offers the public a greater sense of the wrters’ inner lives and provides a resource of wisdom and companionship to other physicians and health care professionals across time and various stages of personal and professional development.

## Notes On Contributors

Dr. Mostwin is Professor at the Brady Urological Institute of the Johns Hopkins Medical Institutions, affiliate faculty of the Berman Institute of Bioethics, and faculty at the Center for Medical Humanities and Social Medicine of the Johns Hopkins University.
