# Supplementary material for: Life-Writing From Medicine: Biographies and Memoirs of Physicians
Source: MedEdPublish (2016). 2018 Sep 5;7:196. [Version 1] doi: 10.15694/mep.2018.0000196.1 (PMC10701819; doi:10.15694/mep.2018.0000196.1)
Supplement: Peer review history

# Reviewer report: Reviewer response for version 1

**DOI:** 10.21956/mep.19652.r29307

**Published:** 2018-09-18

**Authors:** Tim Dornan

**Affiliations:** 1 Queen's University Belfast

This review has been migrated. The reviewer awarded 5 stars out of 5 Life writing, this article tells us, is a type of autobiography that allows authors to share their life experiences with readers at a very personal level. Dr Mostwin’s engaging narrative style illustrates the value of this by giving a taste of many influential physicians’ lives. Medical humanities exercise me because I wonder how many students or doctors have the imagination, and the confidence, to bridge the gap between – let’s say – Elgar’s deeply moving piano quintet and their day-in-day out experiences of medicine. The aesthetic of music resonates with the joy and pathos of clinical practice, but how easily can we bridge the two? Without help, I can’t.To get to the essence of this article, I’d like to quote directly from a paragraph, which made life writing ‘click’ for me. Commenting on books containing physicians’ life writing, Dr Mostwin states: 'It is tempting to acquire these books, categorize them, look for patterns, and generate theories and structures. In medicine, we do that very well. We approach the unknown this way. But already in 1933, Alfred Korzybski, creator of the Theory of General Semantics, had warned us that: "… a map is not the territory”.'If we view medical education as a process of identity development, narrative accounts of individual physicians’ lives offer us possibilities for our own identities. As a medical student in Oxford, I visited the house in Norham Gardens where William Osler had lived but knew or cared little about him. Later in my career, Michael Bliss’s wonderful biography brought the great man to life. This provided me with belated possibilities for my own professional identity. Great lives, this taught me, have a central place in medical education. Dr Mostwin’s article has introduced me to other great lives and, more importantly, to the genre of life writing. This, I am sure, could make important contributions to the identities of future physicians.

Reviewer Expertise:

NA

No decision status is available

# Reviewer report: Reviewer response for version 1

**DOI:** 10.21956/mep.19652.r29309

**Published:** 2018-09-05

**Authors:** John Cookson

**Affiliations:** 1 University of Worcester

This review has been migrated. The reviewer awarded 5 stars out of 5 Thank you; a very helpful review for anyone who plans or is planning the place of medical humanities and especially literature in undergraduate medical education

Reviewer Expertise:

NA

No decision status is available

# Reviewer report: Reviewer response for version 1

**DOI:** 10.21956/mep.19652.r29310

**Published:** 2018-09-05

**Authors:** P Ravi Shankar

**Affiliations:** 1 American International Medical University

This review has been migrated. The reviewer awarded 4 stars out of 5 I enjoyed reading this personal account about the life stories of physicians. Among the books mentioned I have read the books of Dr Varghese, Dr Loxterkamp and Dr Gawande. I and my colleagues had used selections from the book by Dr Loxterkamp during the medical humanities modules we had facilitated at Aruba and Saint Lucia. One of the best advantages of physician life stories is the fact that it provides doctors in training a perspective on the life of a physician. The diverse collection of stories provides a range of experiences which can broaden the education of physicians. The author in the manuscript provides a selection of books by various physician authors which I believe will be of interest to doctors and medical educators and could also be used during medical humanities sessions. I would be interested in knowing details of the conference presentation on which the particular manuscript has been based. I am also happy that the special themed issue on the medical humanities has brought forth such a rich collection of manuscripts.

Reviewer Expertise:

NA

No decision status is available

# Reviewer report: Reviewer response for version 1

**DOI:** 10.21956/mep.19652.r29308

**Published:** 2018-09-05

**Authors:** Trevor Gibbs

**Affiliations:** 1 AMEE

This review has been migrated. The reviewer awarded 3 stars out of 5 I am presuming that this paper was the script of a recent presentation at a conference- it would have been useful to have insight as to which conference this was for referencing purposes and the possibility of whether this talk had been placed on a podcast later.I was fascinated to read this paper and pleased to be given an insight into some books that I had neither seen nor read, as well as being reminded of some almost standard texts.The author mentioned twice in the paper the usefulness of these boos for the public -"the life-writings of physicians also offers the public a greater sense of the writers' inner lives " as well as them also providing "a resource of wisdom and companionship to other physicians and health care professionals across time and various stages of personal and professional development." In this paper though I am not sure that the author provided the answer to his own question- "Where exactly do they fit into literature, into medicine?", and may I add into the development of our new generation of doctors.Although I enjoy reading such papers and like the author fascinated by the stories described, we do have to examine more closely the question of how and where we fit them into medical education in order that we can learn from them and use them to shape this future workforce.

Reviewer Expertise:

NA

No decision status is available
